# Light history modulates growth and photosynthetic responses of a diatom to ocean acidification and UV radiation

**DOI:** 10.1007/s42995-022-00138-x

**Published:** 2022-09-12

**Authors:** Wei Li, Tifeng Wang, Douglas A. Campbell, Kunshan Gao

**Affiliations:** 1 State Key Laboratory of Marine Environmental Science & College of Ocean and Earth Science, Xiamen University, Xiamen, 361005 China; 2 College of Life and Environmental Sciences, Huangshan University, Huangshan, 245041 China; 3 Co-innovation Center of Jiangsu Marine Bio-industry Technology, Jiangsu Ocean University, Lianyungang, 222005 China; 4 Biology Department, Mount Allison University, Sackville, NB E4L 1G7 Canada

**Keywords:** Chlorophyll fluorescence, Growth, Light intensity, Ocean acidification, Pigmentations, UV radiation

## Abstract

To examine the synergetic effects of ocean acidification (OA) and light intensity on the photosynthetic performance of marine diatoms, the marine centric diatom *Thalassiosira weissflogii* was cultured under ambient low CO_2_ (LC, 390 μatm) and elevated high CO_2_ (HC, 1000 μatm) levels under low-light (LL, 60 μmol m^−2^ s^−1^) or high-light (HL, 220 μmol m^−2^ s^−1^) conditions for over 20 generations. HL stimulated the growth rate by 128 and 99% but decreased cell size by 9 and 7% under LC and HC conditions, respectively. However, HC did not change the growth rate under LL but decreased it by 9% under HL. LL combined with HC decreased both maximum quantum yield (*F*_V_/*F*_M_) and effective quantum yield (*Φ*_PSII_), measured under either low or high actinic light. When exposed to UV radiation (UVR), LL-grown cells were more prone to UVA exposure, with higher UVA and UVR inducing inhibition of *Φ*_PSII_ compared with HL-grown cells. Light use efficiency (*α*) and maximum relative electron transport rate (rETR_max_) were inhibited more in the HC-grown cells when UVR (UVA and UVB) was present, particularly under LL. Our results indicate that the growth light history influences the cell growth and photosynthetic responses to OA and UVR.

**Supplementary Information:**

The online version contains supplementary material available at 10.1007/s42995-022-00138-x.

## Introduction

Increasing atmosphere CO_2_ concentration due to human activities since the Industrial Revolution has occurred at a faster rate than in any previous geological period (Hönisch et al. 2012), increasing from a 280 μatm preindustrial level to the present level of ca. 415 μatm; it is expected to reach 1000 μatm within this century (A1Fl scenario) (Gattuso et al. 2015). Increasing concentrations of dissolved anthropogenic CO_2_ in the ocean induces changes in the seawater carbonate system, with a decrease in pH, CO_3_^2−^ and carbonate saturation state, and an increase of CO_2_ and HCO_3_^−^, leading to ocean acidification (OA) (Doney et al. 2009). In parallel, global warming has been suggested to enhance stratification and shoaling of the upper mixed layer, resulting in increased exposure to both visible and UV radiation (UVR) for the phytoplankton within this layer (Gao et al. 2020). As inorganic carbon sources (HCO_3_^−^ and CO_2_) and solar radiation are the principal factors controlling photosynthetic carbon fixation, these expected changes will have profound implications on marine primary production and the marine biological CO_2_ pump.

Phytoplankton in the upper mixing layer often experiences different light intensities and daily dosages as the transmission of visible light and UVR varies spatially and temporally, with generally increasing transmission from coastal waters to the open ocean (Häder and Gao 2015). Moreover, due to differences in mixing rates under future model projected scenarios (Capotondi et al. 2012; Masson-Delmotte et al. 2021), the light intensities to which cells are exposed in the upper mixing layer would be more diverse (Häder and Gao 2015). Cells living under low light levels may occasionally be exposed to UVR due to mixing. While low to moderate levels of UVR are known to sometimes stimulate photosynthetic carbon fixation of coastal phytoplankton (Gao et al. 2007), higher levels of PAR and/or UVR generally show adverse effects (Häder et al. 2015). Therefore, UVR can modulate phytoplankton responses to environmental changes (Jiang et al. 2021; Jin et al. 2017; Li et al. 2012).

A number of studies have documented the effects of OA on growth and physiological performances of marine phytoplankton groups or single species, but findings vary (Beardall et al. 2014) especially when other co-varying ecological drivers are combined (Gao et al. 2019b). The effects of OA on the growth of marine phytoplankton are influenced by PAR levels (Gao et al. 2012b; Li et al. 2014) and are altered by UVR (Boyd et al. 2018; Li et al. 2017; Litchman and Neale 2005). For example, the calcification processes of calcified algae under OA were found to be significantly inhibited by UVR (Gao et al. 2009; Gao and Zheng 2010). Negative effects of UVR on the photosynthetic performance (*F*_V_/*F*_M_) of PS II and relevant proteins (psbD removal rate, ratio of RbcL to psbA) of *Thalassiosira weissflogii* was exacerbated under high CO_2_ and low pH (Gao et al. 2018). However, the evaluation of OA effects normally neglects interacting effects with other key environmental drivers, and how microalgal cells will acclimate and adapt to multiple stressors in future oceans needs to be explored (Boyd et al. 2018; Gao et al. 2019a, b).

Diatoms grown under different light histories may exhibit different responses to OA (Li et al. 2020) and UVR (Guan and Gao 2008). Here, we hypothesize that the low- and high-light growth histories for diatoms may regulate their responses to OA and UVR, and that photosynthetic performances of cells after long term acclimation to low light and lowered pH would be more sensitive to UV exposure.

## Results

### Growth, cell size and pigmentations

**Fig. 1:**
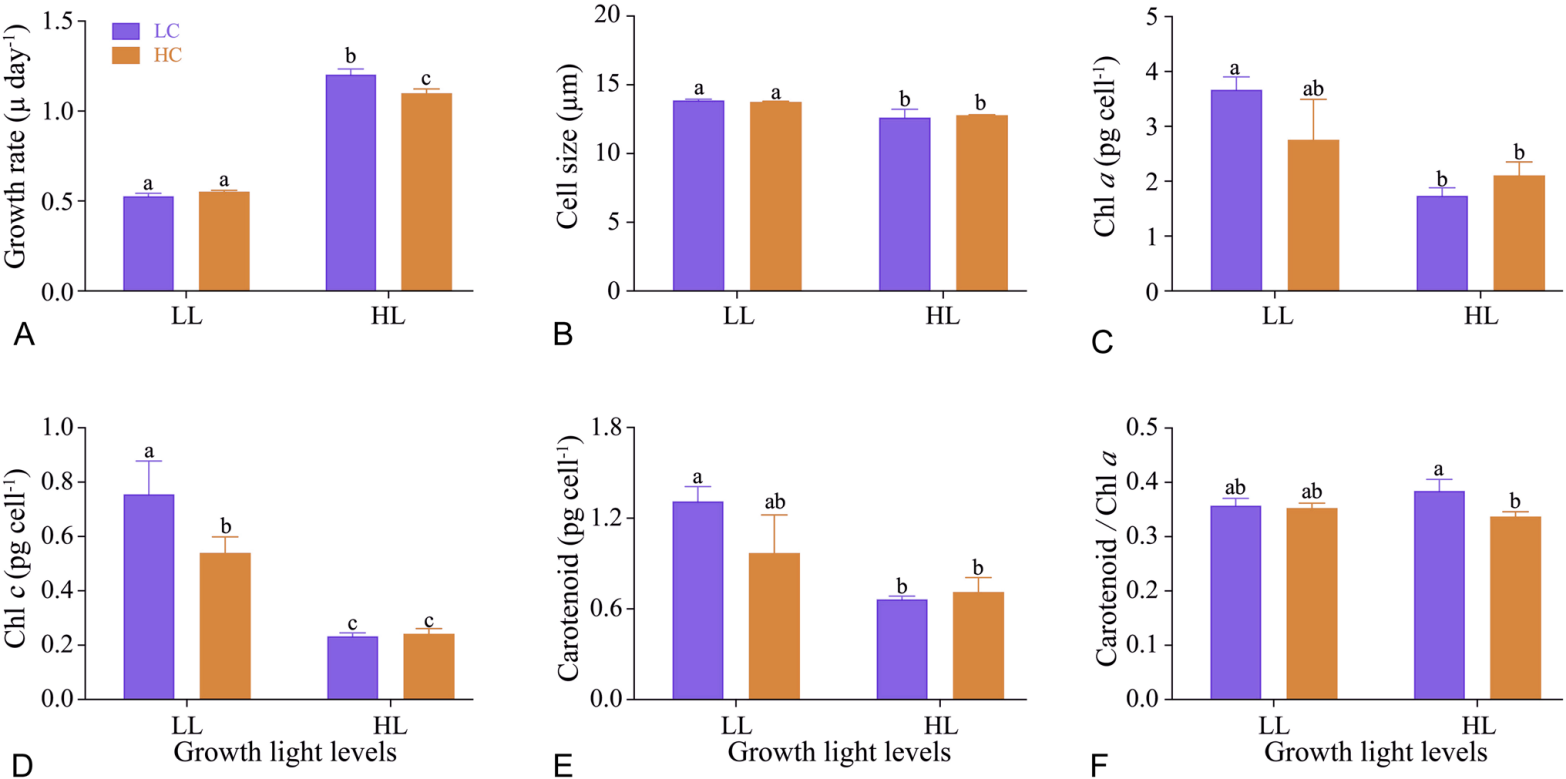
Growth rate (**A**), cell size (**B**), Chl *a* (**C**), Chl *c* (**D**), carotenoid (**E**) and mass ratio of carotenoid to Chl *a* (**F**) of *T. weissflogii* grown under low light (60 μmol m^−2^ s^−1^, LL) and high light (220 μmol m^−2^ s^−1^, HL) levels, with low CO_2_ (390 μatm, LC) and high CO_2_ (1000 μatm, HC) concentrations. Data are represented as means ± SD of triplicate cultures, and different letters above the bar indicate significant difference between treatments (*P* < 0.05)

Based on a two-way ANOVA (all *P* < 0.0001) analysis, it was found that growth rates were significantly affected by light level and CO_2_ both individually and interactively (Table 1). HL stimulated growth rate by 128% under LC and by 99% under HC (all *P* < 0.0001), with no significant change in growth between LC and HC under LL conditions (*P* = 0.559) (Fig. 1A). HC significantly decreased the growth rate by 9% in comparison with LC under HL (*P* = 0.0019) (Fig. 1A). The cell size was also significantly affected by light level (*P* < 0.001) but not by CO_2_ (*P* = 0.87) (Table 1). HL decreased cell size by 9% (*P* = 0.005) under LC and by 7% (*P* = 0.022) under HC compared with growth under LL (Fig. 1B).

**Table 1:** Two or three-way ANOVA analysis of individual and interactive effects of culture light (L) and CO_2_ (C) level and subsequent responses to UVR exposure on the measured parameters of *T. weissflogii*

Parameters	L		C		UVR		L*C		L*UVR		C*UVR		L*C*UVR	
	*F*	*P*	*F*	*P*	*F*	*P*	*F*	*P*	*F*	*P*	*F*	*P*	*F*	*P*
Growth	9.79	**< 0.0001**	9.79	**0.01**	–	–	25.41	**< 0.01**	–	–	–	–	–	–
Cell size	38.78	**< 0.001**	0.03	0.87	–	–	0.71	0.42	–	–	–	–	–	–
Chl *a*	29.28	**< 0.001**	1.29	0.29	–	–	7.26	**0.03**	–	–	–	–	–	–
Chl *c*	1060.00	**< 0.0001**	6.77	**0.03**	–	–	7.99	**0.02**	–	–	–	–	–	–
Carotenoid	29.76	**< 0.001**	3.14	0.11	–	–	5.48	**0.05**	–	–	–	–	–	–
Car/Chl *a*	0.53	0.49	10.24	**0.01**	–	–	6.84	**0.03**	–	–	–	–	–	–
*F*_V_/*F*_M_	0.01	0.99	101.20	**< 0.0001**	–	–	9.53	**< 0.01**	–	–	–	–	–	–
*Φ*_PSII_-CL	70.37	**< 0.0001**	57.33	**< 0.0001**	–	–	6.77	**0.01**	–	–	–	–	–	–
NPQ	13.41	**< 0.001**	2.13	0.17	–	–	0.13	0.88	–	–	–	–	–	–
*Φ*_PSII_-SS	22.51	**< 0.0001**	0.77	0.39	34.29	**< 0.0001**	8.11	**0.01**	8.41	**< 0.001**	1.22	0.32	1.32	0.29
*α*	0.26	0.62	13.55	**< 0.0001**	16.73	**< 0.0001**	6.01	**0.02**	3.52	**0.03**	0.44	0.73	0.66	0.59
rETR_max_	486.05	**< 0.0001**	2.01	0.17	44.78	**< 0.0001**	4.86	**0.03**	1.58	0.21	1.03	0.39	1.06	0.38
*E*_k_	544.55	**< 0.0001**	7.11	**0.01**	45.35	**< 0.0001**	0.01	0.91	2.99	**0.05**	0.36	0.78	0.99	0.41
*InhΦ*_PSII_	16.40	**< 0.001**	5.76	**0.03**	4.70	**0.02**	5.11	**0.03**	3.24	0.06	0.36	0.70	0.39	0.68

Significant difference of treatments was based on the *F* value and subsequent calculated *P* value and indicated with bold font. “–” Not involved; Car/Chl *a*: carotenoid/Chl *a*; *Φ*_PSII_-CL: *Φ*_PSII_-culture light; *Φ*_PSII_-SS: *Φ*_PSII_-solar simulator; *InhΦ*_PSII_: UVR-induced inhibition of *Φ*_PSII_

HL, both individually and interactively with CO_2,_ significantly affected the Chl *a*, Chl *c* and carotenoid concentrations (All *P* < 0.05) (Table 1). There was no significant difference in Chl *a*, Chl *c* and carotenoid between the CO_2_ treatments under either LL- or HL-grown cells (all *P* > 0.05) (Fig. 1C–E), except that Chl *c* decreased by 29% under HC compared to LC under LL (*P* = 0.021) (Fig. 1D). The ratio of carotenoid to Chl *a* did not change at LC compared to HC under LL conditions, however, under HL the ratio of carotenoid to Chl *a* significantly decreased by 12% under HC compared to LC (*P* = 0.014) (Fig. 1F).

### Chlorophyll fluorescence

**Fig. 2:**
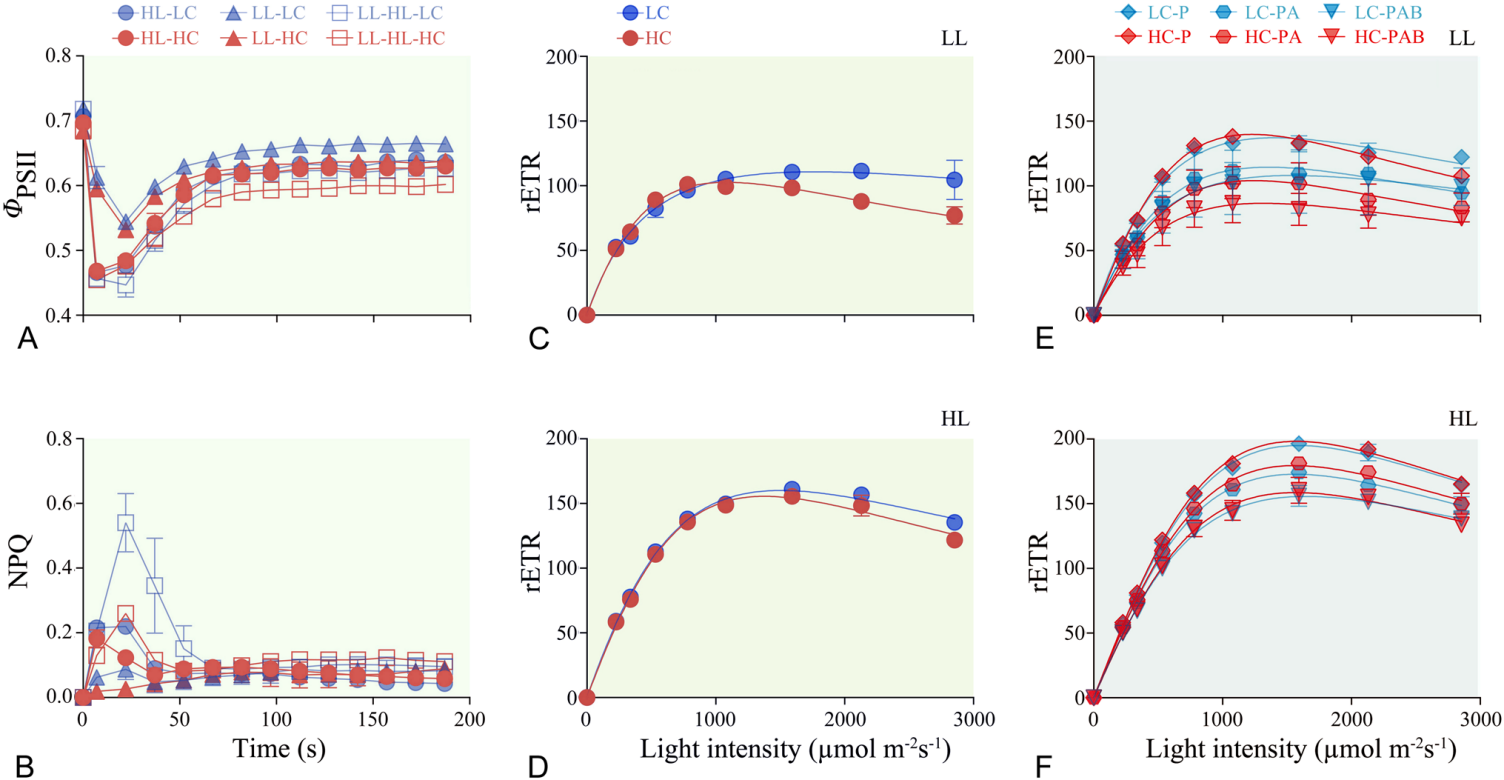
The quantum yield (**A**) and NPQ (**B**) during the induction curve measurements and the rapid light curve (**C**–**F**) of *T. weissflogii* grown under low light (60 μmol m^−2^ s^−1^, LL) (**C**, **E**) and high light (220 μmol m^−2^ s^−1^, HL) (**D**, **F**) with low CO_2_ (390 μatm, LC) and high CO_2_ (1000 μatm, HC) treatments. Induction curve of high-light-grown cells measured with actinic light of 226 μmol m^−2^ s^−1^ (indicate as HL); low-light-grown cells measured with actinic light of 76 μmol m^−2^ s^−1^ (indicate as LL) and 226 μmol m^−2^ s^−1^ (indicate as LL-HL). The rapid light curve of LL- (**C**, **E**) and HL-grown (**D**, **F**) cells were measured from its culture status (**C**, **D**) and after 1 h solar simulator exposure with PAR (P), PAR + UVA (PA) and PAR + UVA + UVB (PAB) (**E**, **F**). Data are represented as means ± SD of triplicate cultures

**Fig. 3:**
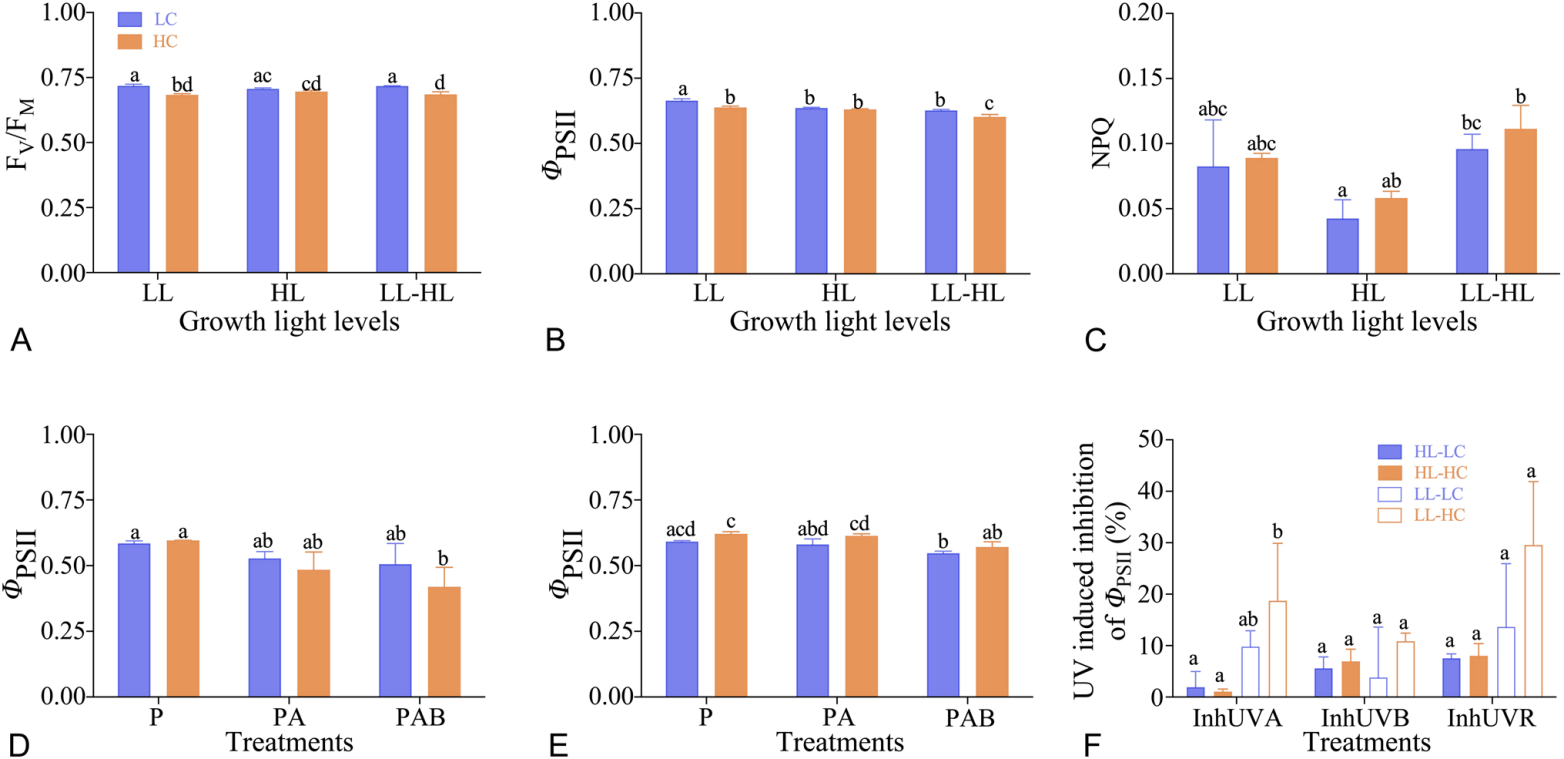
The *F*_V_/*F*_M_ (**A**), *Φ*_PSII_ (**B**) and NPQ (**C**) from last point of the induction curve in Fig. 2, and the measured *Φ*_PSII_ values after exposures under solar simulator to PAR (P), PAR + UVA (PA) and PAR + UVA + UVB (PAB) for 1 h of *T. weissflogii* grown under low light (60 μmol m^−2^ s^−1^, LL) (**D**) and high light (220 μmol m^−2^ s^−1^, HL) levels (**E**), with low CO_2_ (390 μatm, LC) and high CO_2_ (1000 μatm, HC) concentrations. UVA-, UVB- and UVR-induced inhibition of *Φ*_PSII_ are shown in **F**. Data are represented as means ± SD of triplicate cultures, and different letters above the bar indicate significant difference between treatments (*P* < 0.05). LL-HL represents the cells grown under LL and measured at HL

The trends of quantum yield (*Φ*_PSII_) (Fig. 2A) and NPQ (Fig. 2B) derived from the fluorescence induction curves and the rapid light curve (Fig. 2C–F) of *T. weissflogii* grown under different light and CO_2_ were shown in Fig. 2. There were no significant changes in *F*_V_/*F*_M_ and *Φ*_PSII_ between LC and HC in HL-grown cells (all *P* > 0.05) (Fig. 3A, B); however, HC decreased *F*_V_/*F*_M_ (Fig. 3A) and *Φ*_PSII_ (Fig. 3B) in LL-grown cells either measured under low (76 μmol m^−2^ s^−1^) or high (226 μmol m^−2^ s^−1^) actinic light (*P* < 0.05) derived from the induction curve (Fig. 2A). No significant change in NPQ was detected between LC and HC in any treatment (*P* > 0.05) (Fig. 3C). HL treatments did not alter the *F*_V_/*F*_M_, *Φ*_PSII_ or NPQ (Fig. 3A–C) at the same CO_2_ level, except that a decrease of *Φ*_PSII_ under LC conditions was detected (Fig. 3B). *Φ*_PSII_ was significantly affected by culture light level and CO_2_ individually and interactively based on a two-way ANOVA analysis (All *P* < 0.05).

No significant change in *Φ*_PSII_ was observed between LC and HC under either LL (Fig. 3D) or HL treatments (*P* > 0.05) (Fig. 3E), although exposure to UVR significantly decreased the *Φ*_PSII_ (*P* < 0.05) (Fig. 3D, E), except in the LC and LL treatment (*P* > 0.05) (Fig. 3D). By comparison, UVA induced higher inhibition of *Φ*_PSII_ in the LL-grown cells (both LC and HC) compared to UVB (Fig. 3F). The UVR (UVA + UVB)-induced inhibition of *Φ*_PSII_ on the LC and HC cultures were 14 and 30% in LL-grown cells, and 8% under both LC and HC in HL-grown cells (Fig. 3F).

Based on the characteristics of the rapid light curves (Fig. 2C–F; Table 2), significant inhibition of α in HC-grown cells occurred upon UV treatment (PA and PAB treatments) (three-way ANOVA, *P* < 0.0001), as reflected in the interactions of light with CO_2_ (three-way ANOVA, *P* = 0.02) or UVR (three way ANOVA, *P* = 0.03). rETR_max_ of LL-grown cells were more sensitive to UVR treatments (PA and PAB treatments), especially under HC conditions. The culture light intensity, CO_2_ concentration and UVR all showed significant effects on *E*_k_ (three-way ANOVA, all *P* < 0.05) (Table 1). Generally, the UVR-induced reduction of *α*, rETR_max_ and *E*_k_ was more pronounced in HC- and LL-grown cells when the cells were exposed to both UVA and UVB (Table 2).

**Table 2:** The fitted parameters of light using efficiency (*α*), maximum of relative electron transport rate (rETR_max_) and saturation light intensity (*E*_k_) from rapid light curve of Fig. 2

	LL			HL		
	*α*	rETR_max_	*E*_k_	*α*	rETR_max_	*E*_k_
LC						
Initial	0.31 ± 0.06^a^	112.4 ± 6.0^a^	365.5 ± 47.3^a^	0.28 ± 0.02^a^	159.3 ± 3.4^a^	577.8 ± 40.0^a^
P	0.30 ± 0.01^a^	135.8 ± 4.6^a^	452.0 ± 35.7^ab^	0.25 ± 0.01^ab^	196.1 ± 4.8^b^	789.1 ± 47.8^b^
PA	0.24 ± 0.01^a^	114.5 ± 6.6^a^	479.7 ± 2.4^b^	0.25 ± 0.01^b^	173.0 ± 1.4^c^	682.2 ± 27.5^bc^
PAB	0.27 ± 0.03^a^	115.8 ± 25.9^a^	423.8 ± 47.6^ab^	0.23 ± 0.01^b^	158.0 ± 5.0^a^	682.1 ± 49.3^ac^
HC						
Initial	0.28 ± 0.02^a^	102.2 ± 1.5^a^	370.4 ± 26.2^a^	0.26 ± 0.01^a^	158.3 ± 4.8^a^	600.0 ± 25.1^a^
P	0.27 ± 0.00^a^	138.6 ± 2.8^b^	518.3 ± 13.6^b^	0.26 ± 0.00^a^	200.2 ± 3.6^b^	780.9 ± 10.4^b^
PA	0.19 ± 0.03^b^	96.6 ± 14.4^a^	518.1 ± 45.9^b^	0.24 ± 0.00^ab^	181.4 ± 3.3^c^	742.6 ± 20.6^bc^
PAB	0.19 ± 0.02^b^	79.1 ± 14.3^a^	427.4 ± 40.2^ab^	0.22 ± 0.01^b^	160.4 ± 7.7^a^	715.8 ± 35.6^c^

Data are presented as means ± SD based on 3 biological replicates. Different letters indicate significant difference among light treatments (initial: culture light, P: PAR, PA: PAR + UVA, PAB: PAR + UVA + UVB) at a confidence level of 95% (*P* < 0.05)

## Discussion

Our results show that predicted elevated pCO_2,_ with resultant OA, decreased diatom growth rates and the cellular ratio of photoprotection to photosynthetic antenna pigment (carotenoid/Chl *a*) under the growth-saturating light levels (HL). This, however, caused as insignificant change in the growth rate under the growth rate-limiting light levels (LL). OA enhanced the sensitivity of the photosynthetic performance to UVA with exacerbated inhibition of *Φ*_PSII_, rETR_max_, *α* and *E*_k_ under LL.

The effects of OA on marine phytoplankton (diatom, coccolithophores, cyanobacteria etc.) have been well documented [see the reviews by Gao et al. (2019b) and Figuerola et al. (2021) and literature therein]. However, the effects differ due to species-specific energetics and physiological regulations under increased pCO_2_ and decreased pH (Gao et al. 2012a). Elevated pCO_2_ downregulates the energy consuming processes of CO_2_ concentrating mechanisms (CCMs) (Reinfelder 2011), and may favor algal growth; however, the outcome of energy savings depends on the cellular energetic balance over energetic cost against acidic stress, resulting in positive, balanced or negative responses to OA (Gao et al. 2012b). Based on the documented results on the effects of OA on the diatom *Thalassiosira weissflogii*, it appears that different strains, culture conditions (such as the CO_2_ concentration/pH value, light intensity and light quality, photoperiod, temperature, nutrients levels and culturing methods) or acclimation time span (generations) are likely to be responsible for the observed differential responses (Supplementary Table S1). These documented results, together with the present work, further suggest that the effects of OA on diatoms be the result of multifactorial regulations and assessment of OA effects should take in situ environmental conditions into consideration. In this study, OA operated synergistically with HL to decrease the growth rate of *T. weissflogii*, indicating that the energy saved from CCM downregulation under HC conditions might exacerbate photorespiration at high light levels, so that HL–HC-grown cells lost more carbon to cope with the acidic stress, as shown previously in diatoms (Gao et al. 2012b; Qu et al. 2021). 

Different light treatments can modulate morphological features and cellular pigmentations of phytoplankton (Finkel et al. 2010; Li et al. 2020). Changes of cell size may have significant implications on both light (Finkel 2001), nutrients and CO_2_ absorption/diffusion (Armstrong 2008; Flynn et al. 2012) and subsequently influence the metabolic rate and community structure (Finkel et al. 2010; Marañón 2015). A decrease in light-harvesting pigments (both Chl *a* and some carotenoids), with a nearly balanced ratio of carotenoid to Chl *a* in HL-grown cells, reflects a photo-acclimation strategy (Brunet et al. 2011; Janssen et al. 2001; Li et al. 2014, 2020) that prevents over-excitation of PSII electron transport (Gordillo et al. 2003; Xu and Gao 2012). In this study, a decrease in cell size along with decreased pigmentation under HL could have limited light absorption and energy transfer, as pigment-specific light absorption increases as the cell size decreases (Fujiki and Taguchi 2002), giving higher light use efficiency (Jeffrey et al. 1996). Therefore, photophysiological down-regulation together with raised photorespiration and mitochondrial respiration (Gao et al. 2012b; Qu et al. 2021) could be responsible for the observed decrease in the growth rate of HL–HC-grown cells compared to LL–HC-grown ones (Fig. 1A).

The chlorophyll *a* fluorescence of PSII (*F*_V_/*F*_M_) is widely used as a stress indicator in algal physiology study (Beardall et al. 2001; Garcia-Gomez et al. 2014). In the HC treatment, the maximum (*F*_V_/*F*_M_) and effective quantum yield (*Φ*_PSII_) of PSII were lowered under LL but not under HL, indicating that a restricted photon supply, coinciding with changes in pigment composition, must have affected the function of PSII. Although HL apparently compensated for the decrease in *F*_V_/*F*_M_ and *Φ*_PSII_ in the HC-grown cells, it was not sufficient to balance the metabolic costs against the acidic stress associated with OA. Therefore, lowered or enhanced efficiency of energy transfer from photochemistry cannot reflect net biomass build-up or cell growth (Rokitta and Rost 2012).

**Fig. 4:**
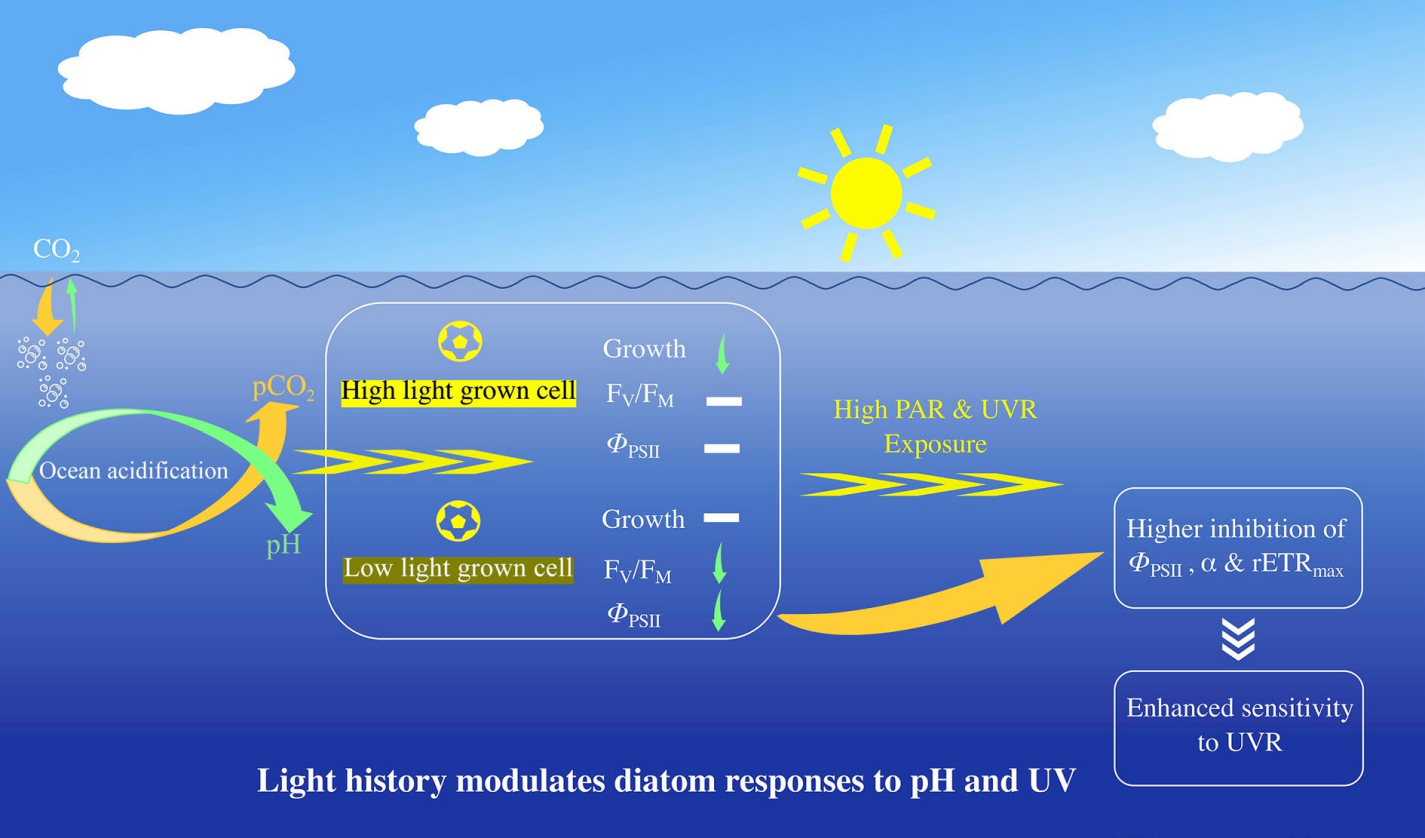
A conceptual diagram illustrating that light history modulates growth and photosynthetic responses of diatom *T. weissflogii* to ocean acidification and UVR

High levels of UVR significantly affect morphological, photophysiological and biogeochemical properties of marine phytoplankton (Häder et al. 2007, 2015). The known effects differ depending upon other covaried environmental drivers. The light history to which cells have been acclimated influences their sensitivity to UVR exposures (Guan and Gao 2008). Compared to cells from shallow or surface layers, harmful effects of UVR on phytoplankton from deeper layers are generally more pronounced (Callieri et al. 2001; Neale et al. 1998). A higher ratio of damage rate to repair rate of PS II and therefore to drops in the abundance of key proteins (for example the psbA protein) and DNA repair upon UVR exposure of LL acclimated cells, may be possible reasons (Crawfurd et al. 2011). Since light saturation intensity of LL-grown cells is lower compared to HL-grown ones, the LL-grown cells were more sensitive to high levels of PAR and UVR. This may be further exacerbated under HC condition as the acidic stress might exacerbate photoinhibition (Wu et al. 2010). OA treatment can exacerbate the stress of UVR on a marine diatom (Gao et al. 2018) and a coccolithophore (Gao et al. 2009). Nevertheless, OA could also eliminate UVR-induced inhibition of photochemical performances, as observed in a green microalga (Garcia-Gomez et al. 2014) and a diatom (Li et al. 2012). Previously, it has been shown that lowered environmental pH together with UVR exposures synergistically enhances synthesis of periplasmic proteins and carbonic anhydrase (Wu and Gao 2009). At lowered pH levels with elevated concentrations of H^+^ in the environment, more protons are transported into the cells (Suffrian et al. 2011); however, the increased levels of carbonic anhydrase and periplasmic proteins can counter this by removing the extra protons to maintain intracellular homeostasis. Subsequently, the acidic stress was lessened and the photochemical performance improved. Here, LL–HC-grown cells were more prone to UVA alone and UVA + UVB-induced inhibition of *Φ*_PSII_, *α*, rETR_max_ and *E*_k_. This signifies that the light history of cells can influence the impacts of OA and UVR on algal photosynthesis (Fig. 4).

Planktonic diatoms living in natural environments experience diverse light environments, varying across days and seasons and modulated by weather conditions and mixing rates in the water column. Since the light history to which diatom cells are acclimated has a significant influence on photosynthetic performance and growth under the influences of OA and UVR, as demonstrated in this work, diatom distributed in different areas and/or depths across different seasons may respond differently to OA and UVR. It should be pointed out that the short-term exposure to UVR in this work does not adequately reflect the performance of diatoms acclimated to solar radiation in the presence of UVR. However, such short exposure to UVR can be considered as a photobiological shock when microalgae from deeper layers are mixed up to surface layer due to perturbations, for example by typhoon-driven mixing. In conclusion, our results highlight the importance of considering the light history of diatoms when assessing their responses to OA and UVR.

## Materials and methods

### Algal culture

The diatom *Thalassiosira weissflogii* (CCMP1336) was acquired from the Center for Collections of Marine Algae (Xiamen University) and grown under cool white-fluorescent lamps at growth limited and saturated light levels (60 μmol m^−2^ s^−1^, low light, LL; 220 μmol m^−2^ s^−1^, high light, HL) under low (390 μatm, low CO_2_, LC, ambient CO_2_ concentration during March to May of 2013) or high CO_2_ (1000 μatm, high CO_2_, HC) conditions. The LL treatments were achieved by covering the culture bottles (polycarbonate, Nalgene, Thermo Scientific) with a neutral density screen. Cells were semi-continuously cultured in bottles at 20 °C with a light:dark cycle of 12:12 (L:D) using artificial seawater enriched with Aquil medium (Morel et al. 1979). The cells in the above treatments were frequently diluted with freshly made medium that were pre-enriched with both LC and HC air using the CO_2_ chamber (HP1000G-D, Ruihua, China). The dilution was carried out every 4 to 5 days, with cell concentration ranging from ca. 50 to 5000 cells ml^−1^, which kept the pH (LL: 8.19 ± 0.01 and 7.87 ± 0.01 in LC and HC; HL: 8.21 ± 0.01 and 7.90 ± 0.02 in LC and HC) and carbonate system stable. Details for controlling the pH and carbonate system in the cultures were summarized in Gao (2021). Cells were grown under different light and CO_2_ condition for ca. 20 generations before being used for subsequent measurements.

### Growth rate and cell size determination

Cell number and cell size were measured with a Z2 Coulter Counter (Beckman, Buckinghamshire, UK). Growth rates (*μ*) were calculated according to the cell density change during a dilution cycle using the equation:

$$\mu =\frac{\mathrm{ln}{N}_{A}-\mathrm{ln}{N}_{B}}{{T}_{A}-{T}_{B}},$$

where *N*_*A*_ and *N*_*B*_ were cell number at time of *T*_*A*_ and *T*_*B*_, respectively.

### Pigmentation determinations

Cells grown for ca. 20 generations under the above CO_2_ and light conditions were sampled onto GF/F filter (Whatman, 0.7 μm) and extracted using methanol (100%) (5 ml) at 4 °C for 12 h. After extraction, the supernatant was acquired by centrifuging the extract at 5000 *g* for 10 min (Universal 320R, Hettich, Germany) and then scanned with a spectrophotometer (DU800, Beckman, California, USA) to obtain optical densities at 470, 632, 653, 665, 666 and 750 nm. The Chl *a* and carotenoid were determined following Ryckebosch et al. (2011) which was modified from Wellburn (1994), and Chl *c* was determined following Ritchie (2006).

### Chlorophyll fluorescence measurements

Chlorophyll fluorescence metrics from time induction curves under a fixed light or from rapid light curves were measured for *T. weissflogii* cells using a Xe-PAM (Walz, Germany). For the measurements of fluorescence induction curves, the saturation light pulse was set at 5000 μmol m^−2^ s^−1^ for 0.8 s, and the LL-grown cells were measured at actinic light of 76 and 226 μmol m^−2^ s^−1^, and for the HL-grown cells it was measured at actinic light of 226 μmol m^−2^ s^−1^. The rapid light curves (RPLs) were measured at actinic lights of 0, 226, 337, 533, 781, 1077, 1593, 2130 and 2854 μmol m^−2^ s^−1^ and then treated with a saturation light pulse of 5000 μmol m^−2^ s^−1^ for 0.8 s after a 10 s exposure under each actinic light level. The *F*_V_/*F*_M_, *Φ*_PSII_ and NPQ under different culture conditions were obtained from the induction curves, and the calculation of parameters followed published equations (Bilger and Björkman 1990; Genty et al. 1989; Kitajima and Butler 1975). The maximum relative electron transport rate (rETR_max_), light use efficiency (*α*) and saturation light intensity (*E*_k_) were determined from RLC fit following Webb et al. (1974).

### Solar UVR exposures

To investigate the photosynthetic responses to short-term UVR of *T. weissflogii* grown under different CO_2_ and light conditions, the cells were transferred into 80 ml quartz tubes and exposed to a solar simulator for 1 h with 580 μmol m^−2^ s^−1^ (ca. 134.5 W m^−2^) of photosynthetically active radiation, 35 W m^−2^ of UVA and 2.5 W m^−2^ of UVB. The levels of PAR, UVA and UVB were chosen according to their daily average values of incident solar radiation in the southern China during summer. Quartz tubes were wrapped with Ultraphan film 395 (UV Opak, Digefra) to acquire PAR exposure (P) only; or with Folex 320 (Montagefolie, Folex, Dreieich, Germany) to filter the UVB range and to acquire PAR + UVA (PA) exposure; or with Ultraphan Film 295 (Digefra, Munich, Germany) to acquire PAR + UVA + UVB exposure (PAB). The intensity of PAR, UVA and UVB were measured with a portable light meter (PMA-2100, Solarlight, USA). During the exposure under the solar simulator, tubes were placed in a temperature-controlled water bath at 20 °C (Eyela, CAP-3000, Tokyo Rikakikai Co. Ltd., Tokyo, Japan). Rapid Light Curves and *Φ*_PSII_ were measured after 1 h exposed to P, PA and PAB.

### Statistics

Data were analyzed using SPSS 19.0 and Prism 9. Two- or three-way ANOVA were used to determine the individual and interactive effects of culture light intensity (low and high light), CO_2_ concentrations (390 and 1000 μatm) and UVR treatments (PAR, PAR + UVA, PAR + UVA + UVB) on the measured parameters, and Tukey’s multiple comparison tests were used to determine differences between treatments at confidence level of 95% (*P* < 0.05).

## Supplementary Information

Below is the link to the electronic supplementary material.

Supplementary file1 (DOCX 35 kb)

## Data Availability

All data generated and analyzed during this study are included in this published article and its additional files.
