# Supplementary material for: Light history modulates growth and photosynthetic responses of a diatom to ocean acidification and UV radiation
Source: Mar Life Sci Technol. 2022 Sep 12;5(1):116–25. doi: 10.1007/s42995-022-00138-x (PMC10077217; doi:10.1007/s42995-022-00138-x)
Supplement: Supplementary file 1 — Supplementary file1 (DOCX 35 kb) [file 42995_2022_138_MOESM1_ESM.docx]

Research paper

**Running title:** Light history modulates diatom responses to pH and UVR

**Light history modulates growth and photosynthetic responses of a diatom to ocean acidification and UV radiation**

Wei Li^1,2^, Tifeng Wang^1^, Douglas A. Campbell^4^, Kunshan Gao^1,3*^

^1^ State Key Laboratory of Marine Environmental Science & College of Ocean and Earth Sciences, Xiamen University, Xiamen 361005, China

^2^ College of Life and Environmental Sciences, Huangshan University, Huangshan 245041, China

^3^Co-Innovation Center of Jiangsu Marine Bio-industry Technology, Jiangsu Ocean University, Lianyungang 222005, China

^4^Biology Department, Mount Allison University, Sackville, NB E4L 1G7, Canada

**Supplementary Table S1** Documented studies on the effects of ocean acidification (OA) on the growth of *Thalassiosira weissflogii* (P, positive; U, unchanged; N, negative; “-”, not available or not shown)

|  | Light intensity (𝛍mol photons m^-2^s^-1^) | Temperature (℃) | L: D | CO_2_ level (𝛍atm) or pH | pH modulation method | Medium | Seawater | Strain | Acclimation generation or days | Growth | Reference |
| --- | --- | --- | --- | --- | --- | --- | --- | --- | --- | --- | --- |
| 1 | 80, 140, 220 | 20 | 12:12 | pCO_2_ (𝛍atm): 410, 1000 | pre-CO_2_ equilibrated medium, semi-continuous culture | Aquil | Natural | CCMP1336 | >10 generations | P, U | Qu et al. (2021) |
| 2 | 100 | 20, 25 | 12:12 | pCO_2_ (𝛍atm): 400, 1000 | pre-CO_2_ equilibrated medium, semi-continuous culture | Modified f/2 | Artificial | CCMA | 339~372 generations | U | Zhong et al. (2021) |
| 3 | 150 | 20 | 12:12 | pCO_2_ (𝛍atm): 25, 50, 100, 200, 400, 800, 1600 | pre-adjusted using closed system approach with HCl, NaHCO_3_ and Na_2_CO_3_ for 25, 50, 100 and 200 𝛍atm, pre-CO_2_ equilibrated medium for 800 and 16**00** 𝛍atm, semi-continuous culture | f/2 | Artificial | CCMA102 | >15 generations | P, U | Li et al. (2019) |
| 4 | 200 | 20 | 12:12 | pCO_2_ (𝛍atm): 400, 1000 | pre-CO_2_ equilibrated medium, semi-continuous culture | Aquil (1/2, 1/5, 1/10 N&P) | Artificial | CCMA102 | >20 generations | U, N | Yang et al. (2018) |
| 5 | Natural solar light | 25-27 | ca. 13:11 | pCO_2_ (𝛍atm): 400, 1000 | pre-CO_2_ equilibrated medium, semi-continuous culture | Aquil (1/2, 3/20 N&P) | Artificial | CCMA102 | >20 generations | P, U, N | (Li et al., 2017) |
| 6 | Natural solar light | - | - | pCO_2_ (𝛍atm): 400, 1000 | Continuously bubbled with target pCO_2_ using pCO_2_ enricher | - | filtered in situ seawater | CCMA102 | 0~33 days | U | (Liu et al., 2017) |
| 7 | 220 | 20 | 24:0 | pCO_2_ (ppmv): 180, 400, 800, 1000 | pre-pCO_2_ equilibrated medium, pH adjusted with HCl and NaOH | Aquil | Natural | CCMP 1336 | < 6 hours | U | (Goldman et al., 2017) |
| 8 | 115 | 20 | 12:12 | steady pCO_2_ (𝛍atm): 400, 1000; fluctuating pCO_2_: 0~870 𝛍atm for LC and 557~1949 𝛍atm for HC | pre-pCO_2_ equilibrated medium, semi-continuous culture | Aquil | Artificial | CCMP 1336 | >15 generations | U | (Li et al., 2016) |
| 9 | 150 | 20 | 12:12 | pCO_2_ (𝛍atm): 386, 614, 795 | pre-pCO_2_ equilibrated medium, semi-continuous culture | Modified Aquil | Natural | CCMP 1336 | 6~7 cell divisions | U | (Sugie and Yoshimura, 2016) |
| 10 | 35, 65 | 15, 20 | 14:10 | pH_Total_:8.6, 8.3 | pre-adjusted using closed system approach with HCl, NaHCO_3_ and Na_2_CO_3_, batch culture | Modified f/2 | Natural | CCMP 1053 | >8 generations | U，N | (Passow and Laws, 2015) |
| 11 | 100 | 15, 20 | 14:10 | pCO_2_ (𝛍atm):400, 1000 | pre-adjusted using closed system approach with NaHCO_3_ and HCl or NaOH, batch culture | Modified f/2 | Artificial | CCMP 1053 | ca.5~6 generations | U, P | (Taucher et al., 2015) |
| 12 | 240 | 16 | 18:6 | pH: 8.8, 8.2, 7.6 | HCl/NaOH, fixed or drift, batch culture over 10 days | Modified Enrichment Solution | Artificial | CCMP 1010 | day0-6, day 8, day 10 | U, N | (Flynn et al., 2015) |
| 13 | 50 | 15, 20 | 14:10 | 15℃: pH_Total_ 7.93~8.21, 7.57~7.76, 7.45~7.66 20℃: pH_Total_ 8.04~8.23, 7.61~7.84, 7.46~7.67 | pre-adjusted using closed system approach with HCl, NaHCO_3_ and Na_2_CO_3_, batch culture | Modified f/2 | Artificial | CCMP 1336 | >8 generations | U | (Shalin et al., 2014) |
| 14 | 240 | 16 | 18:6 | pH: 8.2, 7.6 | HCl/NaOH, fixed or drift, batch culture over 10 days | Modified Enrichment Solution | Artificial | CCMP 1010 | day0-6, day 8, day 10 | U, N | (Clark et al., 2014) |
| 15 | 350 | 20 | 12:12 | pCO_2_ (ppmv): 190, 380, 750 | pre- adjusted to the target pH with HCl and NaHCO_3_ under constant total alkalinity, semi-continuous culture | f/2 | Artificial | CCMP 1336 | >8 cell divisions | P | (Wu et al., 2014) |
| 16 | 80~100 | 20 | 24:0 | pH_Total_: 7.7, 8.1, 8.4 | HCl or NaOH | 100 𝛍mol L^-1^ NO_3_^-^, 10 𝛍mol L-1 PO_4_^3-^, 100 𝛍mol L^-1^ SiO_2_, 1 𝛍mol L^-1^ Fe, various concentrations of Zn | Natural | CCMP 1336 | 7~8 cell divisions | P | (Xu and Gao, 2012) |
| 17 | 200 | 18 | 12:12 | pCO_2_ (ppmv): 150, 280, 380, 770, 1500 | bubbling with target pCO_2_, semi-continuous cultures | Modified Aquil | Artificial | CCMP 1336 | >10 generations | U | (Reinfelder, 2012) |
| 18 | 160 | 20 | 24:0 | pCO_2_ (𝛍atm): 100, 750 | Continuously bubbled with target pCO_2_ using premixed gasses | Aquil | Artificial | Axenic culture, from Provasoli-Giullard Center for the Culture of Marine Protozoa | - | U, N | (Milligan et al., 2009) |
| 19 | 50 | 25 | 24:0 | 5%, 10% and 20% CO_2_ (v/v, in air) | bubbling with target pCO_2_, semi-continuous cultures | f/2 | - | Collected at offshore of Research Institute of Marine Bioresources | 10 days | U, N | (Ishida et al., 2010) |
| 20 | 150 | 15 | 18:6, 24:0 | pH_NBS_: 7.8~9.1 | HCl or NaOH under constant DIC, batch culture | Modified f/2 | Natural | Collected at the Alfred Wegener Institute | >9 generations | P, U | (Burkhardt et al., 1999) |
| 21 | 60, 220 | 20 | 12:12 | pCO_2_ (𝛍atm): 390, 1000 | pre-CO_2_ equilibrated medium, semi-continuous culture | Aquil | Artificial | CCMP1336 | >20 generations | U, N | Present study |

**References**

Burkhardt S, Zondervan I, Riebesell U (1999) Effect of CO_2_ concentration on C:N:P ratio in marine phytoplankton: a species comparison. Limnol Oceanogr 44: 683-690.

Clark DR, Flynn KJ, Fabian H (2014) Variation in elemental stoichiometry of the marine diatom *Thalassiosira weissflogii* (Bacillariophyceae) in response to combined nutrient stress and changes in carbonate chemistry. J Phycol 50: 640-651.

Flynn KJ, Clark DR, Mitra A, Fabian H, Hansen PJ, Glibert PM, Wheeler GL, Stoecker DK, Blackford JC, Brownlee C (2015) Ocean acidification with (de) eutrophication will alter future phytoplankton growth and succession. Proc R Soc Lond B Biol Sci 282: 20142604.

Goldman Johanna AL, Bender Michael L, Morel François MM (2017) The effects of pH and pCO_2_ on photosynthesis and respiration in the diatom *Thalassiosira weissflogii*. Photosynth Res 132: 83-93.

Ishida Y, Hiragushi N, Kitaguchi H, Mitsutani A, Yoshimura M (2010) A highly CO_2_-tolerant diatom, *Thalassiosira weissflogii* H1, enriched from coastal sea, and its fatty acid composition. Fish Sci 66: 655-659.

Li F, Fan J, Beardall J, Xu J (2019) Physiological and biochemical responses of *Thalassiosira weissflogii* (diatom) to seawater acidification and alkalization. ICES J Mar Sci 76: 1850–1859.

Li F, Wu Y, Hutchins DA, Fu F, Gao K (2016) Physiological responses of coastal and oceanic diatoms to diurnal fluctuations in seawater carbonate chemistry under two CO_2_ concentrations. Biogeoscience 13: 6247-6259.

Li W, Yang Y, Li Z, Xu J, Gao K (2017) Effects of seawater acidification on the growth rates of the diatom *Thalassiosira (Conticribra) weissflogii* under different nutrient, light, and UV radiation regimes. J Appl Phycol 29: 133-142.

Liu N, Tong S, Yi X, Li Y, Li Z, Miao H, Wang T, Li F, Yan D, Huang R (2017) Carbon assimilation and losses during an ocean acidification mesocosm experiment, with special reference to algal blooms. Mar Environ Res 129: 229-235.

Milligan AJ, Mioni CE, Morel FMM (2009) Response of cell surface pH to pCO_2_ and iron limitation in the marine diatom *Thalassiosira weissflogii*. Mar Chem 114: 31-36.

Passow U, Laws EA (2015) Ocean acidification as one of multiple stressors: growth response of *Thalassiosira weissflogii* (diatom) under temperature and light stress. Mar Ecol Prog Ser 541: 75-90.

Qu L, Campbell DA, Gao K (2021) Ocean acidification interacts with growth light to suppress CO_2_ acquisition efficiency and enhance mitochondrial respiration in a coastal diatom. Mar Pollut Bull 163: 112008.

Reinfelder JR (2012) Carbon dioxide regulation of nitrogen and phosphorus in four species of marine phytoplankton. Mar Ecol Prog Ser 466: 57-67.

Shalin S, Caitlin F, Ullrich MS, Uta P, William PD (2014) Aggregation and sedimentation of *Thalassiosira weissflogii* (diatom) in a warmer and more acidified future ocean. PLoS ONE 9: e112379.

Sugie K, Yoshimura T (2016) Effects of high CO_2_ levels on the ecophysiology of the diatom *Thalassiosira weissflogii* differ depending on the iron nutritional status. ICES J Mar Sci 73: 680-692.

Taucher J, Jones J, James A, Brzezinski M, Carlson C, Riebesell U, Passow U (2015) Combined effects of CO_2_ and temperature on carbon uptake and partitioning by the marine diatoms *Thalassiosira weissflogii* and *Dactyliosolen fragilissimus*. Limnol Oceanogr 60: 901-919.

Wu Y, Campbell DA, Irwin AJ, Suggett DJ, Finkel ZV (2014) Ocean acidification enhances the growth rate of larger diatoms. Limnol Oceanogr 59: 1027-1034.

Xu J, Gao K (2012) Future CO_2_-induced ocean acidification mediates the physiological performance of a green tide alga. Plant Physiol 160: 1762-1769.

Yang Y, Li W, Li Z, Xu J (2018) Combined effects of ocean acidification and nutrient levels on the photosynthetic performance of *Thalassiosira (Conticribra) weissflogii* (Bacillariophyta). Phycologia 57: 121-129.

Zhong J, Guo Y, Liang Z, Huang Q, Xia J (2021) Adaptation of a marine diatom to ocean acidification and warming reveals constraints and trade-offs. Sci Total Environ 771: 145167.
